# Retention of enzyme activity with a boron-doped diamond electrode in the electro-oxidative nitration of lysozyme

**DOI:** 10.1016/j.enzmictec.2010.02.002

**Published:** 2010-05-05

**Authors:** Jesús Iniesta, María Deseada Esclapez-Vicente, John Heptinstall, David J. Walton, Ian R. Peterson, Victor A. Mikhailov, Helen J. Cooper

**Affiliations:** a Department of Physical Chemistry and Institute of Electrochemistry, University of Alicante, Alicante 03080, Spain; b Centre for Molecular and Biomedical Science, Faculty of Health and Life Sciences, Coventry University, Coventry CV1 5FB, UK; c School of Biosciences, University of Birmingham, Edgbaston, Birmingham B15 2TT, UK

**Keywords:** BDD, Electro-oxidative nitration, Lysozyme, Nitrotyrosine, Tandem mass spectrometry, Infrared multiphoton dissociation

## Abstract

In this paper we report the successful use of a non-metallic electrode material, boron-doped diamond (BDD), for the anodic electro-oxidative modification of hen egg white lysozyme (HEWL). Platinum electrodes can give rise to loss of activity of HEWL in electrosynthetic studies, whereas activity is retained on boron-doped diamond which is proposed as an effective substitute material for this purpose. We also compare literature methods of electrode pre-treatment to determine the most effective in electrosynthesis. Our findings show a decrease in total nitroprotein yield with decreasing nitrite concentration and an increase with increasing solution pH, confirming that, at a BDD electrode, the controlling factor remains the concentration of tyrosine phenolate anion. Purification of mono- and bis-nitrated HEWL and assay of enzymic activity showed better retention of activity at BDD electrode surfaces when compared to platinum. The products from electro-oxidation of HEWL at BDD were confirmed by electrospray ionization Fourier transform ion cyclotron resonance (ESI-FT-ICR) mass spectrometry, which revealed unique mass increases of +45 and +90 Da for the mono- and bis-nitrated lysozyme, respectively, corresponding to nitration at tyrosine residues. The nitration sites were confirmed as Tyr23 and Tyr20.

## 1 Introduction

*In vivo* protein nitration is associated with many disease conditions that involve oxidative stress and the inflammatory response [1,2]. The nitration of protein is taken as a marker of disease; the current evidence does not support its being seen as a causative agent. Proteins can be modified *in vitro* by synthetic electrochemical methods [3]. The advantages over chemical modification are that the protein is maintained in mild conditions throughout electrolysis, the reaction is easily controlled through the applied potential, the reaction may immediately be ceased by switching off the current, there are no chemical residues from reagents and side-products that require complex purification protocols, and the modified protein may be used downstream without separation from the electrolyte.

Electrosynthetic modification of proteins has been shown, under different conditions, to oxidise specific amino acid residues including methionine, tryptophan [4], and tyrosine [5–7]. The selective iodination of horse heart myoglobin has also been described [8]. The electronitration results are in contrast to those achieved by chemical nitration using tetranitromethane [9] or peroxynitrite [10] in which combinations of mono-, bis-, and tris-nitration are observed, and complex separations of products are necessary. Moreover in preparative electro-oxidations it has been shown, by mass spectrometry, that iodination or nitration at tyrosine is the only change occurring in the protein. However, it was not shown unequivocally that there were not more subtle changes that occur, for example as might give rise to conformational shifts that affect bioactivity without altering the mass. In this context, the type of anode material used may affect protein conformation.

Earlier work on the selective electro-oxidative nitration of HEWL was optimised at platinum electrodes, where the selectively formed products were mono- and bis-nitration at Tyr23 and Tyr23 and 20, respectively [7]. However, exposure of the protein to metallic surfaces, particularly Pt or Au, can promote structural and functional changes, and even denaturation to a degree [11]. To overcome this drawback, a non-metallic, highly boron-doped diamond electrode (BDD) might offer some advantage as an alternative to platinum during electrosynthetic modification. Moreover, BDD has a very well known wide potential window in aqueous solution, low background current, and long-term stability. Consequently BDD has been shown a useful electrode material in residual water treatments [12], and in electroanalyses of amines [13] and metals such manganese [14], or sulphur-containing compounds [15]. In addition, these electrodes have been used for the electrochemistry of DNA [16], and in studies focused on the direct electrochemistry of proteins such as myoglobin and haemoglobin [17], blue copper proteins [18] and cytochrome *c* [19]. However, these studies were electroanalytical and involved limited exposure of the protein to the polarised electrode surface, whereas electrosynthetic studies involve exposure of the protein to the polarised electrodes for periods of several hours. It is by no means clear that proteins can withstand this treatment, and the aim of the current work was to directly determine the mass changes (by mass spectrometry) and, indirectly, the conformational changes (by measurement of enzyme activity) occurring during electronitration at a BDD electrode, in comparison to those at a platinum electrode.

## 2 Materials and methods

### 2.1 Chemicals and reagents

Lyophilized hen egg white lysozyme (14.3 kDa, 100,000 U/mg), from Fluka, was used as-received. NaH_2_PO_4_·2H_2_O, was from Fluka; NaNO_2_, Na_2_B_4_O_7_·10H_2_O, H_3_BO_3_ and lyophilized *Micrococcus lysodeikticus* (*luteus*) cell walls were purchased from Sigma–Aldrich. All other reagents were of analytical chemical grade and used without further purification. Double distilled water was filtered in a Millipore Elix 3 system (18 MΩ cm).

### 2.2 Cyclic voltammetric testing of BDD pre-treatment protocols

Cyclic voltammetry was carried out using an Autolab PGSTATS 30 apparatus in an undivided electrochemical cell with a platinum wire as counter electrode and Ag/AgCl/Cl^−^ (3 M) via a Luggin capillary as reference electrode. The working electrode was made-to-measure with a highly boron-doped diamond thin film (1 μm) from the Centre Suisse Electronique et de Microtechnique SA (CSEM), Neuchatêl, Switzerland (1.0 cm^2^, 10 mm × 10 mm, geometric area) with 1200 ppm of boron content. The BDD electrode was attached with silver conductive epoxy to a stainless steel sheet embedded in PTFE with silicon corners covered by a mixture of epoxy-polyurethane DP410 from 3 M. An internal electrical connection was made by the use of a stainless steel rod. All solutions were de-oxygenated by bubbling with argon for 15 min before each experiment.

The working electrode was carefully polished for 4 min before experiments with 1.0 μm alumina and thereafter applying ultrasound from a bath for 1 min in order to remove remaining Al_2_O_3_ particles. The cathodic pre-treatment of the BDD surface was performed by cycling the potential 10 times between 0 and −4 V (vs. Ag/AgCl) at 0.1 V s^−1^ in aqueous 1 M HNO_3_. The anodic pre-treatment followed the same procedure but cycling between 0 and +4 V. Each pre-treatment was repeated until reproducible results were obtained. In all experiments, cyclovoltammograms were performed immediately after surface pre-treatment, thus avoiding the possible modification of consequent surface functional groups by air exposure [20].

### 2.3 Synthetic electro-oxidative nitration of HEWL at a platinum and a BDD electrode

Electrochemical nitration of HEWL at a platinum electrode was carried out using a mesh basket platinum electrode of ca. 30 cm^2^ geometric area. The electrochemical nitration was performed in 50 mM sodium nitrite and 50 mM disodium tetraborate at pH 9.0 with a protein solution of 1 mg mL^−1^, at a fixed potential of +0.85 V vs. saturated calomel electrode (SCE) [7]. The control experiment for exposure of HEWL to a polarised electrode was performed using the same experimental conditions, but in the absence of sodium nitrite.

The electrochemical nitration of HEWL at a BDD electrode was carried out in a water-cooled cell at 284 ± 2 K, fixing the potential at 1.05 V vs. Ag/AgCl/Cl^−^ (3 M) using an Amel Instruments 2053 potentiostat. The cathodic compartment was a 5 mm diameter cylindrical chamber separated from the anodic compartment by a non-selective sintered glass membrane. A stainless steel wire from Goodfellow (2 mm diameter) was used as the counter electrode. The cathodic compartment was filled with 1 mL of buffer solution comprising 50 mM Na_2_B_4_O_7_·10H_2_O and 50 mM NaNO_2_ adjusted to pH within the range of 8.0–10.0 with H_3_BO_3_ or NaOH; for pH 7 50 mM sodium phosphate was used. A 4 cm^2^ geometric area BDD working electrode was pre-treated by electroreduction following the procedure described above and placed near the reference electrode. All of the silicon and stainless steel in the BDD electrode which were in contact with the electroactive solution were covered by a mixture of epoxy-polyurethane DP410 from 3 M. The anodic solution was buffer (50 mL) containing 1 mg mL^−1^ HEWL with different sodium nitrite concentrations ranging from 2 to 100 mM. The working electrode was polished during 4 min and thereafter treated by ultrasonic irradiation in a cleaning bath for 1 min before the cathodic pre-treatment (*vide supra*). Electrosynthetic nitration was monitored by measuring the charge passed with a Digatron electronic A·h coulometer, and the absorbance of the protein solution at 430 nm was measured using a Helios β Unicam UV spectrophotometer. Molar extinction coefficient of nitrotyrosine is 4400 M^−1^ cm^−1^ at 430 nm and pH 10.0 [21].

The reaction products were separated by low pressure cation exchange chromatography, as described previously [5]. After separation, native, mono- and bis-nitrated lysozyme proteins were extensively dialyzed against 10 mM ammonium acetate, pH 6.0, using Spectra/Por molecular porous membrane tubing with 3500 Da cut-off. Samples were subsequently freeze dried. Cell walls from lyophilized *M. lysodeikticus* (0.3 mg mL^−1^ in 0.1 M K_2_HPO_4_, pH 6.2) were used as substrate in a turbidimetric assay for lytic activity of HEWL [5].

### 2.4 Mass spectrometric analysis by ESI-FTICR

Prior to MS analysis, reduction and alkylation of the disulfide bonds in lysozyme samples was carried out as follows: 100 μg of unmodified and nitrated proteins were dissolved in 500 μL buffer solution (0.1 M NH_4_HCO_3_ plus 8 M urea, pH 8.0), and 6.3 μL of a dithiothreitol aqueous solution (DTT), 45 mM, was added to the solutions. The final protein solutions were incubated at 55 °C for 30 min. The reduction mixtures were cooled to room temperature and treated with 6.3 μL of a solution of 63 mM iodoacetamide in 0.1 M NH_4_HCO_3_ (pH 8.0) at room temperature for 1 h. The final alkylated protein solutions were extensively dialysed against 5 mM NH_4_HCO_3_.

All tandem mass spectrometry analysis was performed on a Thermo Finnegan LTQ FT mass spectrometer (Thermo Fisher Scientific, Bremen, Germany). The lyophilised native and nitrated samples (1–3 μM final concentration) were prepared by dissolution in methanol/water (1/1, 1% formic acid), and thereafter introduced into the mass spectrometer by use of an Advion Biosciences Triversa electrospray source (Advion Biosciences, Ithaca, New York). All MS and MS/MS spectra were acquired in the ICR cell with a resolution of 100,000 at *m*/*z* 400. MS/MS spectra were obtained in the ICR cell by infrared multiphoton dissociation (IRMPD) using a 75 W in-built CO_2_ laser (Synrad, Mikilteco, Washington) for 100 ms. Precursor ions for IRMPD were isolated in the linear ion trap (LTQ). Automatic gain control (AGC) target was 2–10 × 10^6^ with maximum fill time 1 s. The isolation width for the protein ions (single charge state) was 20–50 Th. Between 150 and 200 microscans (transients) were averaged for each fragmentation spectrum. Raw MS data were analysed by use of Xcalibur 2.05 software (Thermo Fisher Scientific), where the Xtract program was used for calculating monoisotopic masses (44% fit factor, 25% remainder). ProSight PTM (https://prosightptm.scs.uiuc.edu) was used to search for *b* and *y* protein fragment ions in Single Protein Mode. The mass accuracy for the search was set at 10 ppm.

## 3 Results and discussion

### 3.1 Cyclic voltammetric testing of pre-treatment of a BDD electrode

A number of electrode pre-treatments have been used for BDD in protein electroanalytical studies, and several of these are compared to determine the most effective for electrosynthesis. Simple abrasive cleaning vs. either an anodic or a cathodic pre-treatment are tested.

**Fig. 1:**
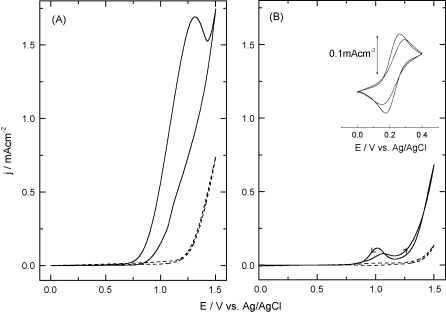
Cyclic voltammograms showing the effect of pre-treatment of the BDD electrode on the electrochemical oxidation of 6 mM NaNO_2_ in 50 mM disodium tetraborate adjusted to pH 9.0 with H_3_BO_3_: (A) cathodic pre-treatment, and (B) anodic pre-treatment. Dotted traces correspond to the background in the absence of nitrite. Inset figure shows the cyclic voltammograms for the electrochemical behaviour of a test redox couple 1 mM K_3_[Fe(CN)_6_] plus 0.1 M KCl in aqueous solution, comparing both electrochemical pre-treatments as described in Section 2: cathodic pre-treatment (solid line) and anodic pre-treatment (dotted line). Cyclovoltammograms were obtained at a scan rate of 0.050 V s^−1^ and the first cycle was recorded.

Fig. 1 shows the effect of the different pre-treatments on the electrochemical oxidation of 6 mM sodium nitrite at a BDD electrode. There are clear differences in peak potential and current density. Cathodic pre-treatment favours a higher current density of 1.7 mA cm^−2^ with a peak potential of +1.32 V. In contrast, cyclic voltammetry of the anodic oxidation of sodium nitrite at platinum electrodes previously showed oxidation of nitrite (in 50 mM sodium tetraborate with 10 mM sodium nitrite [pH 9.4]) at +0.85 V (vs. SCE) with a polycrystalline platinum working anode. Thus, the oxidation potential peak is shifted some +0.5 V for nitrite oxidation at a cathodically pre-treated BDD electrode (+1.32 V vs. Ag/AgCl). The peak potential shift for nitrite oxidation is ascribed to a lower resistivity of the cathodically pre-treated BDD electrode surface [20]. Anodic pre-treatment (Fig. 1B), gave a much lower current density in the positive scan direction for the BDD electrode than was seen following cathodic pre-treatment. Upon abrasion of the electrode with 1 alumina powder/water slurry, a similar voltammetric response was obtained to that from the cathodic pre-treatment. Cathodic pre-treatment is known to produce increased surface conductivity in BDD [22,23], whereas anodic pre-treatment gave effects attributed to surface functionalisation, with the formation of alcohols, ketones and mainly carboxylic groups. In this context, the BDD presents a negatively charged surface at high pH [24], providing a lower current density for the direct electro-oxidation of nitrite. Plots of oxidative peak current *I*_*p*_ vs. the square root of scan rate behave linearly as previously reported for the electrochemical oxidation of nitrite on a BDD electrode [25]. A small non-zero intercept indicates an influence of irreversible kinetic behaviour.

The Fe(CN)_6_^3−/4−^ redox reaction has previously been shown to be sensitive to the nature of a BDD surface [26]. The figure inset in Fig. 1B shows the electrochemical oxidation of 1 mM Fe(CN)_6_^3−/4−^ in aqueous 0.1 M KCl. The cathodic pre-treatment provides a well-defined voltammetric response with a peak-to-peak separation of 83 mV and a ratio of $I_{p}^{c}/I_{p}^{a}$ = 1.0, whereas the anodic pre-treatment has a peak-to-peak separation of 142 mV with a ratio $I_{p}^{c}/I_{p}^{a}$ = 0.89 denoting slower electron transfer kinetics. These results are in agreement with those shown by Marken et al. [19] where a cathodic pre-treatment favours direct electron transfer at BDD electrodes for the ferri/ferrocyanide couple and consequently in electroanalysis of the protein cytochrome *c*. Moreover, peak-to-peak separations for the Fe(CN)_6_^3−/4−^ redox reaction at BDD electrodes are dependent upon pre-treatment nature and duration [20].

Lysozyme at a concentration of either 1 or 5 mg mL^−1^ gave no measurable voltammetric signal at the BDD electrode in 50 mM Na_2_B_4_O_7_ buffer solution at pH 9.0 irrespective of the type of pre-treatment (results not shown). This is not surprising since the enzyme is not redox-active, so the only electroactivity would come from amino acid residues in the protein chain. These are effectively at very dilute concentrations (although discharge of tyrosinylate anions in the chain *is* a component of the nitration mechanism – see results below). However, it is not quite what was found with the platinum electrode where, in cyclic voltammetry, a small anodic current was noted with lysozyme (at 0.85 V vs. SCE) together with evidence for blockage of the electrode by lysozyme, as indicated by the poorly resolved hydrogen and anion adsorption [7]. The BDD electrode, in contrast, with lysozyme at 5 mg mL^−1^ produced an enhancement of circa 300 mV for both oxygen and hydrogen evolution, respectively (*i.e.* a promotion of electrolytic solvent breakdown in the presence of the protein). Moreover the addition of nitrite to protein at either 1 or 5 mg mL^−1^ gave a very well-defined oxidative wave with a peak potential *E*_*p*_ = 1.3 V showing that lysozyme does not participate in blocking of the BDD surface in these conditions.

### 3.2 Preparative electro-oxidative nitration of HEWL at a BDD electrode

**Fig. 2:**
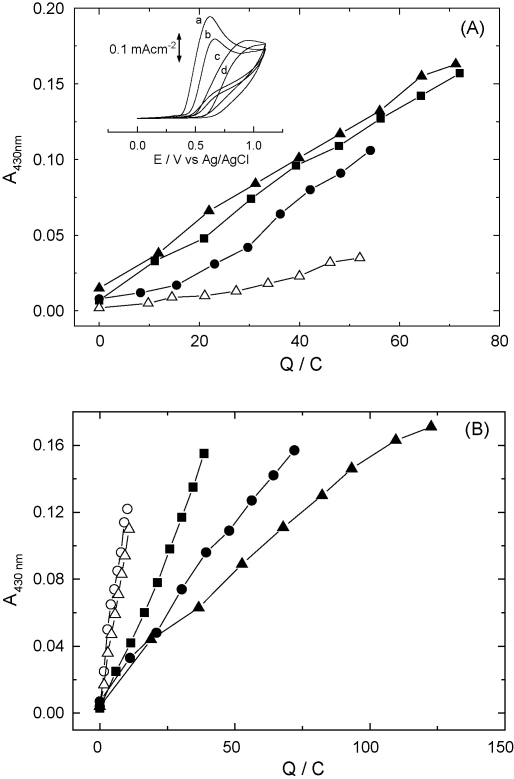
(A) Electrosynthetic nitration of lysozyme at the BDD electrode at varying pH values as a function of charge passed. Lysozyme (1 mg mL^−1^ concentration) was oxidatively nitrated in 50 mM sodium nitrite plus 50 mM disodium tetraborate at pH 10.0 (▴), pH 9.0 (■), or pH 8.1 (●); at pH 7.0 (△) 50 mM sodium phosphate buffer was used. Inset figure shows the cyclic voltammograms (first cycle at 0.050 V s^−1^) for the electrochemical oxidation of 2 mM L-tyrosine at different pH values: (a) pH 10.0, (b) 9.0, (c) 8.0 and (d) 7.0 in the buffer solutions indicated above. (B) Electrochemical nitration of lysozyme (1 mg mL^−1^) at +1.05 V as a function of charge passed using a BDD electrode at different sodium nitrite concentrations of 2 mM (○), 10 mM (△), 20 mM (■), 50 mM (●) or 100 mM (▴). Buffer solutions consisted of 50 mM disodium tetraborate adjusted to pH 9.0 with H_3_BO_3_.

Fig. 2A shows the electrosynthetic nitration of lysozyme at a BDD electrode at varying pH as a function of charge passed. As previously shown with a platinum electrode [7], electro-oxidative nitration is favoured at basic pH, the nitration level plateauing to similar high values at pH values of 9 and 10 (Fig. 2A). However, in the case of tyrosine-containing proteins little clear information has been reported about the reaction mechanisms of oxidation and/or nitration. Ranta et al. [27] reported that between 2 and 3 electrons were released per molecule of tyrosine in peptides when using a glassy carbon electrode. Moreover, the amino acid backbone, peptide composition and the chain length of the peptide can influence the redox properties of the phenolic group of a tyrosyl residue [28,29] leading in general to an oxidative peak at more positive potentials. In proteins, not only the environment of the tyrosine moiety, but also the surface accessibility of the phenolic group may strongly influence the selectivity of reaction. The oxidative peak potential of nitrite on BDD electrodes is pH-independent in the range of pH studied [25], but the protein and tyrosine electro-oxidation are pH-dependent, which is consistent with a reaction mechanism where tyrosylate radical formation is a key step [30] For example, the electro-oxidation of L-tyrosine is pH-dependent (see inset Fig. 2A), showing a decrease of 59 mV per pH unit of the oxidative peak potential for a 10 μМ L-tyrosine concentration. When a higher L-tyrosine concentration was used, the peak potential increased with pH showed non-linear behaviour, probably due to phenomena at the BDD electrode surface.

Fig. 2B shows the evolution of the electrochemical nitration of lysozyme (1 mg mL^−1^) at +1.05 V as a function of charge passed for different sodium nitrite concentrations. The lower sodium nitrite concentrations provide a much higher current efficiency, displaying a similar behaviour for 2 and 10 mM nitrite. We note that for a same electrolysis time there was only a roughly 2-fold increase in the nitration level of HEWL when comparing the 2 mM and 100 mM nitrite concentrations (*i.e.* over a 50-fold increase in nitrite concentration). The higher level of nitration is favoured as nitrite concentration increases. Moreover, this results in a shorter exposure of the enzyme at the electrode, and therefore authors speculate a diminution of the electrode/protein interaction.

### 3.3 Yields of mono- and bis-nitrated tyrosine as a function of nitrite concentration and pH

The products separated by ion exchange chromatography were mono- and bis-nitrated lysozyme as previously demonstrated when using platinum (as foil or basket) or copper as electrode materials [5,7]. In these earlier papers we also demonstrated that mononitration of tyrosine 23 was followed by bis-nitration at tyrosines 23 and 20, with negligible tris-nitration involving tyrosine 53, which destroys enzyme activity. In the current study, even at longer oxidation times, there was negligible formation of a tris-nitrated lysozyme. Tyrosine 53 in HEWL is largely buried in a cleft and thereby the electro-oxidative nitration of Tyr53 is almost negligible, consistent with the assumption that the tyrosyl radical is produced by a direct electron transfer process. This is an important feature of the electrosynthetic nitration, since chemical nitrating agents tend to give a product mixture which includes detrimental nitrotyrosinylation at position 53.

Table 1 presents the results of varying nitrite concentration on the HEWL peak ratios of mono-, bis- and un-nitrated forms after 4 h of electronitration on a cathodically pre-treated BDD electrode at a fixed protein concentration and pH value. The significant observation is that a 50-fold increase in nitrite concentration increases nitrotyrosine yields some 7.5-fold, though the ratio of mononitro to bisnitro HEWL peak moves to favour the bisnitro with increasing nitrite concentration. Thus the higher relative yield of mononitrotyrosinylated product is obtained at the lower (≥10 mM) nitrite concentrations. The nitrite concentration certainly affects the accumulated passage of charge, showing that nitrite discharge does occur in the system.

Table 2 shows results from ion exchange chromatography after 4 h of electronitration of HEWL at a cathodically pre-treated BDD electrode (+1.05 V) at variable pH and with fixed concentrations of nitrite (at 50 mM) and of protein. At pH values of 7 and 8 the formation of nitrated lysozyme is less favoured than at higher pH. The ratio of mono- to un-nitrated HEWL remains relatively constant over the pH range of 7–8, while at higher pH values bis-nitration is favoured. Charge passage and total absorbance at 430 nm both plateau between pH 9 and 10. Note that the area of this BDD electrode is very much smaller than that of the platinum basket used in previous work which limits the extent of the electro-oxidation, thus this passage of charge represents only fractional reaction.

The low levels of mono- and bis-nitrated lysozyme formation may be a result of the instability and decomposition of NO_2_ to nitrite and nitrate ions, or the NO_2_ radical concentration and the probability of coupling NO_2_ and tyrosyl radicals in proteins for the formation of 3-nitrotyrosine. Moreover, the pH influences the yields of mono- and bis-nitrated lysozyme. The fact that yields for the electronitration of lysozyme are low as pH decreases, might be related to a change in the direct electron transfer potential of lysozyme to more positive values, which is in turn dependant upon the extent of deprotonation of the tyrosine hydroxyl group. This has also been demonstrated by the electro-oxidative behaviour of tyrosine-containing peptides [29].

### 3.4 Mass spectrometry

Electrospray ionisation Fourier transform ion cyclotron resonance (ESI-FTICR) mass spectrometry was used to evaluate protein mass changes to indicate covalent modification upon electronitration, and infrared multiphoton dissociation (IRMPD) of the intact protein ions was carried out in the ICR cell to confirm the sites of nitration.

**Fig. 3:**
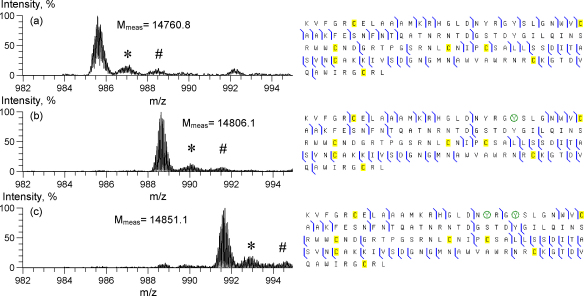
MS and MS/MS data for (A) unmodified, (B) mono- and (C) bis-nitrated HEWL protein. Disulfide bonds were reduced and alkylated prior to the analysis. Left column – ESI mass spectra of 15+ charge state, right column – IRMPD fragmentation diagrams. The major peaks in the mass spectra correspond to protonated ions, and are labelled with the values of their measured monoisotopic masses *M*_meas_ (Da). (*) and (#) labels indicate peaks corresponding to adduction of one and two sodium ions, respectively. Other minor peaks could not be identified with certainty. First nitration is assumed to take place at Tyr23 (see text).

MS and MS/MS data for unmodified and nitrated HEWL with disulfide bonds alkylated prior to MS analysis are shown in Fig. 3. Xtract analysis of protein masses reveals the characteristic shift of +45 and +90 Da from the monoisotopic mass of the protein from the control sample (calculated *M* = 14,760.1 Da) for mono- and bis-nitrated lysozyme, respectively (Fig. 3B and C). No mass shift was found for the MS peak of lysozyme exposed to an electro-oxidising potential of 1.05 V in the absence of nitrite (data not shown). Therefore HEWL protein remains chemically stable when in contact with either electrode material. Summaries of IRMPD fragments observed are given in the right column of Fig. 3. Total number of *b* and *y* fragments is 50, 49 and 47 for unmodified, mono- and bis-nitrated HEWL, respectively. The nitration site in the mono-nitrated lysozyme was assumed to be at Tyr23. However, as no IRMPD cleavages between Tyr20 and Tyr23 have been observed for mono-nitrated HEWL, our IRMPD data do not exclude the possibility of alternative mono-nitration at Tyr20. For bis-nitrated lysozyme IRMPD produces several *b* fragments including one between Tyr20 and Tyr23. These fragments indicate the presence of nitration on both tyrosines. Furthermore, there are 10 *b* fragments containing nitro groups between Tyr23 and Tyr53 in each of the fragmentation diagrams for mono- and bis-nitrated lysozyme, which indicate that Tyr53 is *not* nitrated during the electrochemical process. This expected result also emphasises the stability of HEWL during electro-oxidative nitration on BDD electrode.

### 3.5 Cell wall turbidimetric assay for HEWL

A comparative study of the cell wall turbidimetric assay of control, oxidised and nitrated lysozymes prepared at either the platinum basket or a BDD electrode was performed, as shown in Table 3. Specifically the ‘control’ sample was recovered from a solution mixture with electrodes inserted that had been prepared for electrolysis but which had not been exposed to a polarising potential; the ‘oxidised’ sample had been exposed to the relevant oxidising potential, but in the absence of nitrite; while the ‘unreacted’ lysozyme was recovered after being present throughout an electrolysis (so it had undergone long-term exposure to a polarised electrode in the presence of the full reaction mixture). A clear and evident result from this study is that lysozyme exposed to the platinum basket electrode loses activity, and the consequent nitrated products lose even more activity. In contrast lysozyme and products exposed to the BDD electrode lose significantly less of their activity, even though the appropriate passage of charge at the BDD electrode requires exposure times of several hours. We point out that this platinum basket was chosen for its high surface area leading to a rapid generation of nitrated product, in contrast to the small surface area of the earlier-studied platinum foil electrodes.

The exposure of proteins to metallic surfaces may cause denaturation to a greater or lesser degree. It has been shown that protein adsorption is affected by the oxidation state or other effects at a metal surface [31]. At gold electrodes, lysozyme adsorption produces surface-induced conformational changes, which decrease as the polarity of the surface increases [32]. It has been known for a long time that proteins adsorb, and may polymerise, on metal electrode surfaces [33]. However, BDD presents an inert surface, resulting in resistance to deactivation of activity [34,35]. We have no evidence for any covalent modification, other than nitration, at either a platinum or a BDD electrode. However, on mononitration, activity is lost with platinum but not BDD (Table 3), therefore nitration per se does not bring about loss of activity. It can be concluded therefore that exposure of lysozyme to platinum does bring about some change in conformation, not mass, that is reflected in loss of activity. Lysozyme nitrated at BDD shows retention of activity of 96% for the mono-nitrated product and 87% for the bis-nitrated derivative, as opposed to platinum where the corresponding figures are 39% and 13%. When retention of protein conformation in nitration is important therefore, use of the BDD electrode is to be preferred over the use of platinum.

There are significant applications in the labelling, immobilisation and biosensing of proteins as well as the production of novel proteins for studies involving pathophysiological dysfunctions in oxidative stress.

## Figures and Tables

**Table 1:** Results of ion exchange chromatographic analysis after 4 h electronitration of lysozyme (1 mg mL^−1^) at a BDD electrode as a function of sodium nitrite concentration. 4 cm^2^ electrode area. 50 mM disodium tetraborate pH 9.0 (adjusted with boric acid). The potential was fixed at +1.05 V vs. Ag/AgCl. Temperature: 284 K.

Constant protein concentration at fixed pH	Variable sodium nitrite concentration				
	2 mM	10 mM	20 mM	50 mM	100 mM
Ratio of mono-nitrated to native lysozyme, measured at 280 nm, per charge passed /C	2.08	2.17	7.58	12.99	24.14
Ratio of bis-nitrated to native lysozyme, measured at 280 nm, per charge passed /C	2.00	1.86	7.41	6.10	7.58
Ratio of mono- to bis-nitrated lysozyme measured at 280 nm per charge passed/ C	1.04	1.17	1.03	2.13	3.18

Accumulated charge passed (*Q*) after 4 h of electronitration / C	10.8	10.2	38.6	72.0	122.6

**Table 2:** Results of ion exchange chromatographic analysis after 4 h electronitration of lysozyme (1 mg mL^−1^) at a BDD electrode as a function of pH. 4 cm^2^ electrode area. 50 mM disodium tetraborate pH 9.0 (adjusted with boric acid) and 50 mM sodium nitrite. Potential was fixed at +1.05 V vs. Ag/AgCl. Temperature: 284 K.

Constant protein concentration at fixed nitrite concentration, pH variable	pH 7.0	pH 8.0	pH 9.0	pH 10.0
Ratio of mono-nitrated to native lysozyme, measured at 280 nm, per charge passed /C	6.37	6.94	12.85	9.01
Ratio of bis-nitrated to native lysozyme, measured at 280 nm, per charge passed /C	0.694	1.04	6.10	4.57
Ratio of mono- to bis-nitrated lysozyme, measured at 280 nm, per charge passed /C	9.18	6.73	2.11	1.97

Accumulate charge passed (*Q*) after 4 h of electronitration / C	52.1	54.2	72.0	71.2

**Table 3:** Cell wall turbidimetric assay for the measurement of lytic activity comparing electro-oxidative nitrations of HEWL protein at platinum and BDD electrodes. Lysozyme activity unit definition: one unit of lysozyme activity will produce a decrease in absorbance at 450 nm of 0.001 per minute in the experimental conditions. Number of trials = 5, 95% confidence interval of the mean. Temperature = 298 K.

	Native lysozyme recovered from an electrolysis solution without polarisation of the electrode	Lysozyme exposed to polarised electrode without nitrite	‘Unreacted’ lysozyme recovered after electrolysis	Mono-nitrated lysozyme from electrolysis	Bis-nitrated lysozyme from electrolysis
Cell wall lytic activity (min^−1^ mg^−1^) Pt basket electrode	30,233 ± 2033	24,267 ± 733^a^	22,267 ± 833^b^	11,817 ± 300	4067 ± 533

Cell wall lytic activity (min^−1^ mg^−1^) BDD electrode	27,667 ± 339	25,424 ± 2034^c^	28,167 ± 995^d^	26,467 ± 1409	24,205 ± 1658

a 0.85 V vs. SCE.

b 0.85 V vs. SCE.

c 1.05 V vs. Ag/AgCl.

d 1.05 V vs. Ag/AgCl.
